# Public Awareness of Obesity as a Risk Factor for Cancer in Central Saudi Arabia: Feasibility of ChatGPT as an Educational Intervention

**DOI:** 10.7759/cureus.50781

**Published:** 2023-12-19

**Authors:** Turki M Alanzi, Wala Alzahrani, Nouf S Albalawi, Taif Allahyani, Atheer Alghamdi, ‏Haneen Al-Zahrani, ‏Awatif Almutairi, Hayat Alzahrani, Latifah Almulhem, Nouf Alanzi, Abdulrhman Al Moarfeg, ‏Nesren Farhah

**Affiliations:** 1 Department of Health Information Management and Technology, College of Public Health, Imam Abdulrahman Bin Faisal University, Dammam, SAU; 2 Department of Clinical Nutrition, College of Applied Medical Sciences, King Abdulaziz University, Jeddah, SAU; 3 College of Medicine, Tabuk University, Tabuk, SAU; 4 College of Applied Medical Sciences, Umm Al-Qura University, Makkah, SAU; 5 ‏Collage of Pharmacy, Taif University, Taif, SAU; 6 ‏Department of Hematology, ‏Armed Forces Hospital at King Abdulaziz Airbase Dhahran, Dhahran, SAU; 7 Department of Clinical Laboratories Sciences, College of Applied Medical Sciences, Jouf University, Jouf, SAU; 8 College of Pharmacy, Taif University, Taif, SAU; 9 College of Medicine, King Faisal University, Al Hofuf, SAU; 10 College of Science and Arts, King Khalid University, Muhayil Asir, SAU; 11 Department of Health Informatics, College of Health Sciences, Saudi Electronic University, Riyadh, SAU

**Keywords:** overweight, obesity, knowledge, public, cancer, awareness, artificial intelligence, chatgpt

## Abstract

Background: While the link between obesity and chronic diseases such as diabetes and cardiovascular disorders is well-documented, there is a growing body of evidence connecting obesity with an increased risk of cancer. However, public awareness of this connection remains limited.

Study purpose: To analyze public awareness of overweight/obesity as a risk factor for cancer and analyze public perceptions on the feasibility of ChatGPT, an artificial intelligence-based conversational agent, as an educational intervention tool.

Methods: A mixed-methods approach including deductive quantitative cross-sectional approach to draw precise conclusions based on empirical evidence on public awareness of the link between obesity and cancer; and inductive qualitative approach to interpret public perceptions on using ChatGPT for creating awareness of obesity, cancer and its risk factors was used in this study. Participants included adult residents in Saudi Arabia. A total of 486 individuals and 21 individuals were included in the survey and semi-structured interviews respectively.

Results: About 65% of the participants are not completely aware of cancer and its risk factors. Significant differences in awareness were observed concerning age groups (p < .0001), socio-economic status (p = .041), and regional distribution (p = .0351). A total of 10 themes were analyzed from the interview data, which included four positive factors (accessibility, personalization, cost-effectiveness, anonymity and privacy, multi-language support) and five negative factors (information inaccuracy, lack of emotional intelligence, dependency and overreliance, data privacy and security, and inability to provide physical support or diagnosis).

Conclusion: This study has underscored the potential of leveraging ChatGPT as a valuable public awareness tool for cancer in Saudi Arabia.

## Introduction

Obesity has emerged as a significant global public health concern, affecting millions of individuals across diverse age groups and backgrounds. The detrimental consequences of obesity on physical health are well-documented, ranging from an increased risk of cardiovascular diseases, diabetes, and musculoskeletal problems [1-4]. However, a less recognized but equally critical aspect of obesity is its association with cancer. Several studies have elucidated a strong correlation between excess body weight and the development of various types of cancers [5-8]. There exists a significant association between obesity and various prevalent forms of cancer, such as breast, colorectal, esophageal, kidney, gallbladder, uterine, pancreatic, and liver cancer. The presence of obesity is associated with an elevated risk of mortality due to cancer and has the potential to impact the selection of treatment options. Approximately 4-8% of cancer cases can be attributable to obesity [9]. For instance, in the postmenopausal stage, obese or overweight individuals are 1.2 to 1.4 times more likely to get breast cancer; and are 1.2 times more likely to get the condition for every 5-unit increase in body mass index (BMI) [10,11]. In the premenopausal stage, obese or overweight individuals are 0.8 times more likely to get breast cancer [11,12]. Furthermore, obese individuals are 1.3 times more likely to get colorectal cancer. Similarly, people with severe obesity are seven times more likely to get endometrial cancer [13,14]. 

Research into cancer risk awareness in different regions has shown differences in understanding between groups based on factors such as socio-economic status and ethnicity [15]. A recent study in Pakistan [16] has found that most of the women lacked knowledge of breast cancer, its symptoms, and its screening tools for early detection. Similarly, in Malaysia [17], issues such as low rates of cancer screening and awareness, delays in seeking medical attention, slow detection and diagnosis times, and insufficient access to high-quality care have led the country's cancer survival rates to fall below the global average. In the UK, public knowledge of the risks of obesity and associations with cancer was found to be less, as only 10.1% in 2014 [18], 15.4% in 2019 [18], and 16% in 2023 [19] of the total population are aware that obesity or overweight is a risk factor for cancer. Although studies conducted in Saudi Arabia have identified good levels of general knowledge of cancer among the public, the knowledge of risk factors such as obesity, overweight, and diet is considerably low [20-22]. Breast, prostate, lung, and colorectum cancers are a few prevalent types of cancers in Saudi Arabia [23]. The epidemiology of certain cancers in Saudi Arabia has exhibited a threefold increase in scale in recent years, reflecting the diversity of cancer types prevalent in the country. The observed rise in cancer incidence can potentially be linked to several factors, including the shifting lifestyle patterns within the Saudi population, wherein a Western model is increasingly being adopted. Additionally, inadequate cancer awareness, the absence of comprehensive screening and early detection initiatives, and prevailing social barriers to cancer investigations may also contribute to this increase. The identified risk factors for cancer in Saudi Arabia include obesity, genetic predisposition, a sedentary lifestyle, tobacco use, viral infections, as well as deficiencies in iodine and vitamin D [24].

Nevertheless, there is a limited body of research investigating the association between variations in BMI and the level of awareness regarding the correlation between overweight/obesity and the risk of developing cancer. Furthermore, there is a limited body of research that has investigated the association between individuals' awareness of cancer risk and their weight, specifically in relation to whether they have received guidance from healthcare experts regarding the significance of keeping a healthy body weight. Prior studies have indicated that overweight and obese individuals tend to express a greater desire to reduce their weight and make efforts to engage in weight loss strategies following recommendations from healthcare professionals [25]. However, it is worth noting that a small proportion of patients indicate that they have been provided with guidance regarding weight reduction [25]. There is a shared sentiment of irritation among both patients and healthcare providers when it comes to engaging in discussions about weight loss guidance [26]. Healthcare workers may lack the necessary training and knowledge [27,28] to effectively communicate with patients regarding the significance of weight as a critical risk factor for many health disorders, such as cancer [29]. Therefore, there is a need for effective educational interventions integrated with innovative technologies such as Artificial Intelligence (AI) to raise public awareness about cancer and its risk factors [30]. AI tools such as ChatGPT operate as a sophisticated language model leveraging a transformer architecture, specifically GPT (Generative Pre-trained Transformer). Trained on a diverse corpus of text from the internet, it comprehends and generates human-like text responses based on the input it receives. Its capabilities encompass a broad spectrum of tasks, including language translation, text completion, question answering, summarization, and conversation. By understanding context, semantics, and patterns in the input text, ChatGPT generates coherent and contextually relevant responses, drawing from its vast knowledge base. It employs deep learning techniques to predict and generate text sequences, offering assistance, information, or engaging in conversation across various topics and domains, making it a versatile and powerful tool for natural language understanding and generation [30]. Recent studies [31-34] have found that ChatGPT proved to be effective in increasing public awareness levels of different types of cancers.

In Saudi Arabia [35], as in many parts of the world [4], the prevalence of obesity has surged over the past few decades. This rising trend in obesity is accompanied by an increasing burden of cancer within the region. Despite the known link between obesity and cancer, there exists a noticeable gap in public awareness regarding this relationship, as well as strategies to mitigate associated risks. Public education is a cornerstone of preventive healthcare, and the dissemination of knowledge regarding the obesity-cancer connection is of paramount importance. Therefore, this study explores an innovative approach to address this gap in knowledge and public awareness of overweight/obesity as a risk factor for cancer and analyzes public perceptions on the feasibility of ChatGPT, an artificial intelligence-based conversational agent, as an educational intervention tool. 

This study holds immense regional relevance. Central to this significance is the cultural fabric and lifestyle intricacies unique to this region, influencing perceptions and behaviors concerning health. Understanding the specific healthcare landscape and epidemiological patterns in this area enables tailored interventions aligned with available resources. By pinpointing region-specific trends in obesity and cancer awareness, this study facilitates gaining insights crucial for crafting targeted educational programs and policies that resonate with the local population. Such focused interventions are pivotal in improving awareness levels and fostering healthier lifestyle choices tailored to Central Saudi Arabia's societal and healthcare dynamics.

## Materials and methods

The present study used a mixed method approach: deductive quantitative cross-sectional approach to draw precise conclusions based on empirical evidence on public awareness of the link between obesity and cancer; and inductive qualitative approach to interpret public perceptions on using ChatGPT for creating awareness of obesity, cancer, and its risk factors [36].

Study setting and participants

This study explicitly focused on the public in Saudi Arabia; therefore, all the residents living in Saudi Arabia who are aged 18 years or above were included in the study. All individuals aged below 18 years are excluded from the study. The survey data was collected using an online survey platform. Interviews with participants were conducted online using Zoom application.

Questionnaire design

The survey questions were derived from a prior study conducted by Hooper et al. [37] in the UK. Supplementary elements were integrated from alternative survey instruments and modified as deemed appropriate. New questions were designed and assessed for their clarity, content, and question style in situations where no pre-existing tools were available.

The study investigated the level of awareness regarding the risk of cancer by employing two types of questions: an unprompted open-ended question inquiring about health disorders associated with obesity or being overweight, and a prompted question asking about specific health conditions that can arise from obesity or being overweight. The response options provided were diabetes, heart disease, stroke, cancer, and arthritis. Both unprompted and prompted cancer knowledge were categorized as dichotomous variables, specifically indicating whether individuals were aware of obesity as a risk factor for cancer or not. The participants were presented with a comprehensive inventory comprising 13 distinct cancer types. They were thereafter instructed to indicate whether an individual's likelihood of having each cancer would be heightened as a consequence of being overweight or obese. The survey participants were given response options of 'Yes', 'No', and 'Don't know'. For the purpose of analysis, the researchers selected four cancer kinds that are commonly observed in Saudi Arabia and are known to be connected with body weight. These cancer types are breast cancer (specifically in postmenopausal women), lung cancer, colorectal cancer, and prostate cancer. The responses were categorized into two variables: 'Yes' indicating that participants had chosen the proper response, and 'No' indicating that they had mistakenly picked the cancer kind. Responses indicating a lack of knowledge were recategorized as negative responses, and ‘do not know’ options. A pilot study was conducted with 13 randomly selected participants from online health communities. The Cronbach's alpha coefficient for the questionnaire was found to be 0.913, suggesting that the questionnaire responses had a high level of internal consistency [38].

Recruitment and sampling

The data was collected through an online survey questionnaire and online semi-structured interviews. The survey link and invitation to participate in interviews was forwarded to the public in Saudi Arabia through emails, various social media platforms, and online health communities. The participants were recruited using convenience sampling techniques from various online communities.

Given the population in Saudi Arabia to be 37 million [39], the estimated sample size was calculated using Cochran's formula [40], which is identified to be 394, and the post-hoc power analysis resulted in 100% power.

Data collection

As stated earlier, the survey link was forwarded to 628 phlebotomists. At the end of four weeks after forwarding the link, a total of 521 responses were collected. However, 35 responses were not fully completed. After removal of incomplete responses, a total of 486 responses were considered for the data analysis. About 148 participants agreed to participate in the interviews. As only those participants who were aware of ChatGPT were considered for interviews, 21 individuals participated in the online interviews.

Ethics

Before commencing the survey, informed consent was obtained from each participant after they had been fully apprised of the study's objectives. The anonymity of the participants and their rights about the data were protected. All the ethical procedures prescribed at the Imam Abdulrahman Bin Faisal University were followed. Ethical approval for the study was obtained from the Research Ethics Committee at the university (approval IRB-2023-03-476).

Data analysis

In order to analyze the overall survey data, statistical techniques including means, relative frequencies, and standard deviations were used as the majority of the data was numerical. Additionally, one-way ANOVA and t-tests were performed using Statistical Package for Social Sciences (SPSS) (IBM Corp., Armonk, NY, USA) to compare the significant statistical differences between participant groups. To analyze interview data, thematic analysis was used.

## Results

Survey results analysis

Table 1 showcases a demographic overview of respondents based on several variables. Gender distribution reveals a slight dominance of males (55.3%) over females (44.7%).

**Table 1:** Participants demographics

Variables		N	Relative frequency
Gender	Male	269	55.3%
	Female	217	44.7%
Age (in years)	18-30	226	46.5%
	31-40	171	35.2%
	41-50	75	15.4%
	>=51	14	2.9%
Education	Uneducated	22	4.5%
	Primary/secondary education	44	9.1%
	Diploma	188	38.7%
	Bachelor’s degree	127	26.1%
	Master’s degree	32	6.6%
	Others	73	15.0%
Socio-economic status	AB	182	37.4%
	C1	94	19.3%
	C2	31	6.4%
	DE	179	36.8%
Region	Central	100	20.6%
	Northern	124	25.5%
	Eastern	114	23.5%
	Southern	59	12.1%
	Western	89	18.3%
Body Mass Index (BMI)	Underweight	40	8.2%
	Normal weight	196	40.3%
	Overweight	158	32.5%
	Obese	79	16.3%
Cancer diagnosis	Ever been diagnosed with cancer	55	11.3%
	Never been diagnosed with cancer	431	88.7%

In terms of age groups, the majority falls within the 18-30 bracket (46.5%), followed by 31-40 years (35.2%), with smaller percentages in the 41-50 years (15.4%) and >=51 years (2.9%) categories. Education-wise, the largest portion holds a diploma (38.7%), followed by bachelor's degrees (26.1%), while uneducated (4.5%) and primary/secondary-educated individuals (9.1%) form smaller segments. Socio-economic status shows a varied distribution, with AB (37.4%) and DE (36.8%) comprising the most substantial proportions. Regionally, Northern (25.5%) and Eastern (23.5%) areas have relatively higher representation compared to Central (20.6%), Southern (12.1%), and Western (18.3%) regions. The BMI categories depict a significant number in the normal weight range (40.3%), followed by overweight (32.5%), while underweight (8.2%) and obese (16.3%) individuals form smaller sections. Finally, the data illustrates that 11.3% of respondents have been diagnosed with cancer, while the majority (88.7%) have not received such a diagnosis.

About 65% of the participants are not completely aware of cancer and its risk factors. Table 2 outlines the mean ratings of awareness concerning cancer across various participant groups based on different variables. Gender-wise, both males (mean = 1.98) and females (mean = 1.96) exhibit similar levels of awareness, with no statistically significant difference observed (p = .4179). However, significant differences in awareness were observed concerning age groups (p < .0001), socio-economic status (p = .041), and regional distribution (Central: p = .0351). Specifically, participants aged 18-30 demonstrated low awareness (mean = 2.06), while those aged 41-50 showed comparatively higher awareness (mean = 1.85). Moreover, individuals belonging to the AB socio-economic group (mean = 1.94) and the Central region (mean = 1.88) displayed higher awareness levels compared to other socio-economic groups and regions, respectively. Education level, BMI, and cancer diagnosis status did not show statistically significant differences in awareness levels among the participants. The standard deviations associated with the mean ratings suggest relatively consistent levels of variance within the groups, indicating the consistency of awareness ratings within each category.

**Table 2:** Differences between participant groups in relation to cancer awareness SD: Standard deviation; *: Statistically significant difference at .05 CI; Mean: (out of 3 ratings: 1: yes; 2: no; 3: Do not know)

Variables		N	Mean	SD	P-value
Gender	Male	269	1.98	0.14	.4179
	Female	217	1.96	0.12	
Age (in years)	18-30	226	2.06	0.13	< .0001*
	31-40	171	1.91	0.12	
	41-50	75	1.85	0.12	
	>=51	14	1.96	0.06	
Education	Uneducated	22	1.97	0.11	.5471
	Primary/secondary education	44	2.05	0.08	
	Diploma	188	1.98	0.16	
	Bachelor’s degree	127	1.93	0.10	
	Master’s degree	32	1.97	0.15	
	Others	73	1.96	0.12	
Socio-economic status	AB	182	1.94	0.11	.041*
	C1	94	1.92	0.13	
	C2	31	2.04	0.17	
	DE	179	2.02	0.13	
Region	Central	100	1.88	0.11	.0351*
	Northern	124	1.97	0.13	
	Eastern	114	2.03	0.13	
	Southern	59	2.00	0.15	
	Western	89	1.98	0.10	
Body Mass Index (BMI)	Underweight	40	1.97	0.12	.2594
	Normal weight	196	2.04	0.12	
	Overweight	158	1.95	0.14	
	Obese	79	1.98	0.12	
Cancer diagnosis	Ever been diagnosed with cancer	55	1.98	0.16	.9262
	Never been diagnosed with cancer	431	1.97	0.12	

Table 3 presents data on participants' awareness concerning the link between cancer and overweight/obesity across various demographic variables. About 72.1% of participants are not completely aware of the link between cancer and overweight/obesity. Overall, the mean ratings indicate relatively consistent levels of awareness among different participant groups, with no statistically significant differences observed in most categories. Gender-wise, both males (mean = 1.48) and females (mean = 1.47) demonstrated similar levels of awareness, with no significant disparity (p = .7593).

**Table 3:** Differences between participant groups in relation to awareness of link between cancer and overweight/obesity SD: Standard deviation; *: Statistically significant difference at .05 CI; Mean: (out of 3 ratings: 1: yes; 2: no; 3: Do not know)

Variables		N	Mean	SD	P-value
Gender	Male	269	1.48	0.04	.7593
	Female	217	1.47	0.05	
Age (in years)	18-30	226	1.47	0.04	.4572
	31-40	171	1.47	0.04	
	41-50	75	1.47	0.04	
	>=51	14	1.39	0.05	
Education	Uneducated	22	1.52	0.04	.2879
	Primary/secondary education	44	1.47	0.04	
	Diploma	188	1.47	0.05	
	Bachelor’s degree	127	1.48	0.04	
	Master’s degree	32	1.51	0.03	
	Others	73	1.42	0.04	
Socio-economic status	AB	182	1.47	0.04	.8009
	C1	94	1.49	0.05	
	C2	31	1.48	0.03	
	DE	179	1.47	0.04	
Region	Central	100	1.48	0.05	.0562
	Northern	124	1.47	0.04	
	Eastern	114	1.43	0.04	
	Southern	59	1.51	0.03	
	Western	89	1.50	0.04	
Body Mass Index (BMI)	Underweight	40	1.48	0.04	.8703
	Normal weight	196	1.48	0.04	
	Overweight	158	1.46	0.04	
	Obese	79	1.49	0.04	
Cancer diagnosis	Ever been diagnosed with cancer	55	1.47	0.04	.9841
	Never been diagnosed with cancer	431	1.47	0.04	

Similarly, age groups, education levels, socio-economic status, BMI categories, and cancer diagnosis status did not exhibit statistically significant differences in awareness regarding the link between cancer and overweight/obesity. However, a marginal trend is noticeable in certain categories. For instance, participants aged >=51 years showed slightly higher awareness (mean = 1.39) compared to other age groups, although this difference was not statistically significant (p = .4572). Likewise, individuals in the "Others" category of education (mean = 1.42) and those in the Eastern region (mean = 1.43) displayed slightly higher awareness levels, but these differences did not reach statistical significance (p = .2879 for education; p = .0562 for region). While there were minor fluctuations in awareness levels across some categories, the data overall suggests a relatively uniform level of awareness regarding the link between cancer and overweight/obesity among the surveyed groups, with no statistically significant differences observed in most cases.

Table 4 compares participant groups' awareness regarding the link between overweight/obesity and four specific types of cancer: breast, lung, colorectum, and prostate. The data showcases varying levels of awareness across demographic categories for each cancer type. For breast cancer, both genders displayed moderate awareness without a significant difference between males (mean = 1.44) and females (mean = 1.38). Across age groups, individuals aged >=51 years showed comparatively lower awareness (mean = 1.21), although not statistically significant. Similarly, no significant differences in awareness were observed concerning education level, socio-economic status, region, BMI, or cancer diagnosis status for breast cancer. For lung cancer awareness, again, gender, age, education, socio-economic status, region, BMI, and cancer diagnosis did not show significant differences in awareness levels among participant groups. Overall, the data suggests relatively consistent awareness levels across these demographic categories regarding the link between overweight/obesity and breast and lung cancers, with no statistically significant disparities observed.

**Table 4:** Differences between participant groups in relation to awareness of link between cancer and overweight/obesity and four cancer types SD: Standard deviation; *: Statistically significant difference at .05 CI; Mean: (out of 3: rating 1: yes; rating 2: no)

Variables		N	Breast		Lung		Colorectum		Prostate	
			Mean	P-value	Mean	P-value	Mean	P-value	Mean	P-value
Gender	Male	269	1.44	.1765	1.57	.9812	1.45	.999	1.47	.7058
	Female	217	1.38		1.58		1.43		1.49	
Age (in years)	18-30	226	1.42	.0886	1.58	.6301	1.47	.2381	1.47	.8547
	31-40	171	1.37		1.56		1.46		1.51	
	41-50	75	1.51		1.60		1.36		1.47	
	>=51	14	1.21		1.43		1.29		1.43	
Education	Uneducated	22	1.50	.1638	1.45	.3813	1.45	.8966	1.59	.2015
	Primary/secondary education	44	1.36		1.66		1.41		1.48	
	Diploma	188	1.46		1.60		1.47		1.43	
	Bachelor’s degree	127	1.40		1.54		1.42		1.57	
	Master’s degree	32	1.47		1.63		1.47		1.50	
	Others	73	1.29		1.51		1.41		1.44	
Socio-economic status	AB	182	1.37	.3886	1.52	.1513	1.40	.3123	1.54	.1566
	C1	94	1.40		1.66		1.50		1.49	
	C2	31	1.48		1.58		1.52		1.42	
	DE	179	1.45		1.58		1.45		1.43	
Region	Central	100	1.39	.0524	1.62	.0689	1.48	.0583	1.45	.3991
	Northern	124	1.39		1.53		1.44		1.53	
	Eastern	114	1.33		1.51		1.37		1.48	
	Southern	59	1.53		1.71		1.59		1.39	
	Western	89	1.49		1.57		1.40		1.53	
Body Mass Index (BMI)	Underweight	40	1.38	.3392	1.57	.4935	1.44	.2841	1.51	.2321
	Normal weight	196	1.37		1.54		1.46		1.54	
	Overweight	158	1.42		1.54		1.41		1.48	
	Obese	79	1.53		1.68		1.58		1.35	
Cancer diagnosis	Ever been diagnosed with cancer	55	1.45	.4925	1.6	.6563	1.47	.6313	1.41	.304
	Never been diagnosed with cancer	431	1.40		1.56		1.41		1.49	

In relation to obesity management, the majority of the participants considered that it is the responsibility of individuals (88.3%), followed by the Ministry of Health (82.7%), parents (78.5%), schools (77.2%), and government (52.7%).

**Figure 1:**
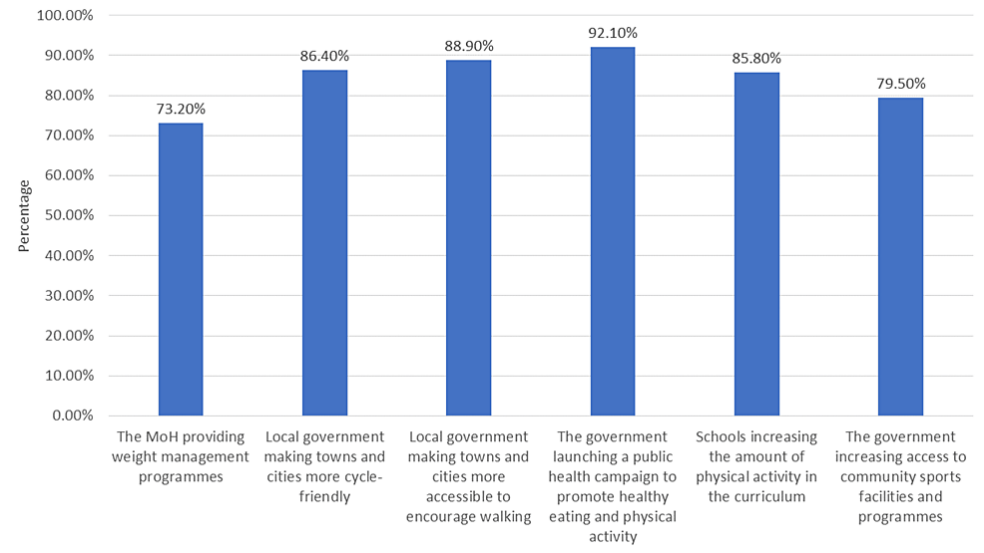
Top ways to reduce prevalence of obesity as suggested by participants MoH: Ministry of Health

The data from Figure 1 showcases varying levels of support among survey participants for different strategies aimed at reducing obesity prevalence. Notably, there's robust support (above 85%) for initiatives such as making towns and cities more accessible for walking, launching public health campaigns promoting healthy habits, and increasing physical activity within school curricula. Additionally, there's considerable backing (around 79.50%) for the government's efforts to enhance access to community sports facilities. However, comparatively lower support (73.20%) is indicated for the Ministry of Health's direct provision of weight management programs. These findings suggest a collective preference for holistic approaches, emphasizing environmental changes, education, and awareness campaigns to combat obesity.

Interview results

A total of 10 themes were analyzed from the interview data, which included five positive factors (accessibility, personalization, cost-effectiveness, anonymity and privacy, multi-language support) and five negative factors (information inaccuracy, lack of emotional intelligence, dependency and overreliance, data privacy and security, and inability to provide physical support or diagnosis). Most of the interviewees (19/21) observed that ChatGPT can be accessed 24/7, as per their convenience, at any time from any location, giving them the freedom to learn according to their lifestyles. Furthermore, almost 50% of the interviewees (10/21) observed that ChatGPT could provide personalized information and advice based on their queries and concerns, catering to specific needs and situations. Most of the participants (17/21) observed that ChatGPT is effective for creating awareness as it can be freely accessed (version 3.5), and there was no need for the government to invest huge amounts in health awareness campaigns. One of the key advantages observed by a few interviewees (6/21) is the anonymity and privacy. They were more comfortable asking sensitive questions or seeking advice on health concerns due to the anonymity provided by interacting with ChatGPT, thus preserving their privacy. Most of the participants (20/21) observed that they were very comfortable with ChatGPT as they could interact with it in Arabic and in English.

In relation to negative aspects, half of the interviewees (11/21) observed that the application was not able to respond to some of their queries, stating that it was not updated with the latest information, while a few interviewees (4/21) observed some inaccuracies in the information when they cross-checked the information with their physicians. Few participants (3/21) observed that the application responses in Arabic lacked emotional appeal. They observed that the application lacks emotional intelligence and may not fully understand or address the emotional needs of individuals dealing with health concerns. Some interviewees (8/21) observed a serious risk that users might overly rely on ChatGPT for health-related advice, potentially delaying seeking professional medical help when necessary. In addition, few participants (7/21) raised data privacy and security concerns, as they share their personal information, and this information may be misused. Also, its inability to effectively diagnose or provide physical support was identified to be the drawbacks of ChatGPT.

## Discussion

The purpose of this study is to analyze public awareness of overweight/obesity as a risk factor for cancer and analyze public perceptions on the feasibility of ChatGPT as an educational intervention tool. Accordingly, the survey results revealed that only 35% had general awareness of cancer and 28.8% were aware that overweight/obesity could be a risk factor for various cancer types, which is very low compared to 57.5% awareness levels among the UK population [37]. Misconceptions regarding the association between overweight and obesity and various forms of cancer were prevalent among the participants. Higher levels of awareness were observed for cancers affecting organs of the digestive system, such as the colon and kidney. However, there was no significant increase in awareness of tumors affecting reproductive systems, such as the womb or postmenopausal breast. Such misconceptions need to be addressed by creating awareness among the public through healthcare professionals and educational interventional tools. It was also observed that there were statistically significant differences among the participant age groups, socio-economic status, and region in relation to general cancer awareness but no differences were observed among the participants groups in relation to awareness of the link between overweight/obesity and cancer. These findings indicate the need to strategize intervention tools according to the population. For instance, high unawareness levels were observed among uneducated, non-working and non-skilled working class compared to educated, upper-middle and skilled working class participants. Therefore, the intervention tools for creating awareness must be cost-effective, easily accessible, supportive of local language and culture to support specific groups like uneducated or less educated individuals. Also, there is a need to expand the research in this area especially the awareness of obesity as a risk factor for cancer, as the prevalence of obesity is rapidly increasing in Saudi Arabia and at a global level. Although there are previous studies that explored general cancer awareness [20-22] and awareness of obesity as a cancer risk factor [37,41], there is limited data available assessing the differences among the public categorized by various factors such as socio-economic status, and particularly data exploring knowledge of risks related to specific cancer types. This study has addressed this gap by providing awareness across cancer types including breast (44.2% awareness), lung (36.4%), colorectum (46.3%), and prostate (32.9%) cancer types. This indicates the lack of knowledge among the public on how obesity and overweight are associated with different types of cancers. To address this situation, the need for creating public awareness through physicians and effective technological intervention tools such as AI chatbots were suggested in various studies [37,42-47].

The existing misconceptions regarding the association between overweight/obesity and different types of cancer may arise from a variety of sources. First and foremost, public education and awareness initiatives may not adequately prioritize the connection between obesity and cancer. Many educational campaigns prioritize addressing broader health issues associated with obesity, such as cardiovascular illnesses or diabetes, while neglecting its association with specific types of cancer. Furthermore, the intricate nature of scientific facts and medical research may not be adequately conveyed to the general populace. It may be difficult to fully understand and spread knowledge about the complex molecular processes that connect fat to the development of cancer. Moreover, errors may be influenced by cultural or societal beliefs surrounding body weight and health. The prevailing notion that obesity is mainly linked to lifestyle choices or aesthetics, rather than being a significant risk factor for cancer, may contribute to these misunderstandings. To correct these misunderstandings, it may be necessary to implement focused and culturally responsive educational initiatives. These interventions should provide clear and easily understandable information about the connection between obesity and different forms of cancer. This will help improve the public's comprehension and awareness.

Accordingly, the findings from interviews suggested that AI application ChatGPT can be an effective intervention tool for creating awareness, especially considering the differences of awareness among the participants in different age groups, social-class, and regions. It is cost-effective, Arabic language supportive, can be accessed at any time from any place and any device. However, primary concerns include data security and privacy, which can significantly influence its adoption as educational interventional tool. Therefore, the government and relevant institutions must formulate regulations and standards on reporting guidelines to strengthen data security and privacy protocols and to address users' concerns about sharing personal health information on AI applications like ChatGPT. Few other recommendations to use ChatGPT as cancer awareness tool include: a) Provide healthcare professionals with ongoing training and updates about ChatGPT's capabilities and limitations. Encourage them to recommend ChatGPT as a supplementary educational tool to patients seeking information about cancer and related health issues; b) Implement interactive educational campaigns utilizing ChatGPT on various social media platforms and healthcare websites. Engage users through quizzes, interactive Q&A sessions, and informative content to increase awareness and knowledge about cancer risks; c) Establish a system to monitor and evaluate ChatGPT's effectiveness regularly. Collect feedback from users to assess their satisfaction, comprehension of information, and any areas that require improvement; d) Support public awareness programs or initiatives led by healthcare authorities in Saudi Arabia by integrating ChatGPT as an educational tool.

Implications

The incorporation of the outlined recommendations into the study focusing on utilizing ChatGPT as a public awareness tool for cancer in Saudi Arabia carries significant practical and theoretical implications. Practically, the implementation of updated content accuracy, multilingual support, personalized responses, and emotional intelligence integration within ChatGPT can profoundly impact the dissemination of accurate and culturally sensitive information. This approach addresses the diverse informational needs of the Saudi Arabian population, fostering more informed decision-making regarding cancer risks, symptoms, and available treatments. Moreover, the emphasis on security measures and continuous training for healthcare providers amplifies trust and reliability in ChatGPT, encouraging its use as a supplementary educational resource.

Theoretically, integrating these recommendations underscores the evolution of AI-driven platforms, demonstrating the potential of ChatGPT not just as an information provider but also as an empathetic and culturally aware conversational agent. Tailoring information based on individual concerns aligns with the principles of personalized medicine, emphasizing the importance of catering to diverse health needs. Additionally, the collaboration with healthcare institutions and the incorporation of interactive educational campaigns represents a novel integration of AI technology into public health initiatives, highlighting its potential as a complementary tool in healthcare settings. By monitoring and evaluating ChatGPT's effectiveness, this study contributes to the growing body of research on AI's role in public health communication, emphasizing the need for ongoing improvements and user-centered design in such applications. Overall, these practical and theoretical implications reinforce ChatGPT's potential as a valuable and adaptable tool for fostering cancer awareness and health literacy in Saudi Arabia and potentially in similar socio-cultural contexts globally.

Limitations

Despite its comprehensive approach, this study exploring the use of ChatGPT as a public awareness tool for cancer in Saudi Arabia faces several limitations. Firstly, the assessment of ChatGPT's effectiveness primarily relies on user feedback and perceptions, potentially introducing biases based on individual experiences or expectations. Moreover, the study's scope might not encompass the entirety of Saudi Arabia's diverse population, potentially overlooking regional variations in language, cultural nuances, or varying levels of technological access. Additionally, the reliance on existing data and algorithms within ChatGPT poses a limitation, as the platform's responses are based on previously learned information, which might not always reflect the most current or locally relevant data. Furthermore, the study's duration might limit the understanding of long-term user engagement and the evolving nature of user needs, preferences, and conversational patterns over time. These limitations underscore the need for future studies to address these gaps, considering a more diverse population, exploring real-time data integration, and conducting longitudinal assessments to better comprehend the sustained impact and effectiveness of ChatGPT as an educational tool for cancer awareness in Saudi Arabia.

## Conclusions

In conclusion, this study has underscored the potential of leveraging ChatGPT as a valuable public awareness tool for cancer in Saudi Arabia. Through the implementation of user-friendly interfaces, tailored content, and language customization, ChatGPT has demonstrated its ability to engage users effectively, disseminate accurate information, and address queries regarding cancer-related concerns. The findings illuminate the positive reception and perceived usefulness of ChatGPT among Saudi Arabian users, highlighting its role in complementing traditional awareness campaigns and bridging informational gaps. However, this study acknowledges the limitations in its approach, such as the reliance on user feedback, potential biases, and constraints related to the platform's capabilities. To maximize the efficacy of ChatGPT as a public health tool, future endeavors should strive to address these limitations by integrating real-time data, expanding the study's scope to encompass a more diverse population, and conducting longitudinal assessments to ascertain sustained impact and adaptability over time. Ultimately, ChatGPT stands as a promising avenue in augmenting cancer awareness initiatives in Saudi Arabia, warranting further exploration and refinement to optimize its utility and reach.
